# The Present-Day Treatment of Laryngeal Tuberculosis

**Published:** 1908-11-21

**Authors:** Harold Barwell

**Affiliations:** Surgeon for Diseases of the Throat, St. George's Hospital, Laryngologist to the Mount Vernon Consumption Hospital, Consulting Surgeon for Throat and Ear Diseases to the Cripples' Home for Girls


### Hospital Clinics.

### THE PRESENT-DAY TREATMENT OF LARYNGEAL TUBERCULOSIS.

By HAROLD BARWELL, M.B.Lond., F.R.C.S.Eng., Surgeon for Diseases of the Throat, St. George's Hospital, Laryngologist to the Mount Vernon Consumption Hospital, Consulting Surgeon for Throat and Ear Diseases to the Cripples' Home for Girls.

Before Krause and Heryng in 1885 and 1887 first advocated the use of lactic acid and the curette in the treatment of laryngeal tuberculosis, it was generally considered that this affection was hopelessly incurable, the beginning of a not distant end, and that little could be done to relieve the pain of the unfortunate patient except to give increasing doses of morphia and cocaine. Consequently, when these methods of treatment were introduced, they were adopted with enthusiasm—an enthusiasm which was at times too indiscriminate, and many patients in an advanced stage of cachexia were subjected to a routine and purposeless course of clippings and cauterisations which only increased their misery. Then came the inevitable reaction, and active treatment was, at least in this country, condemned in all cases by a great number of laryngologists. Other causes contributed to this result; improved methods in the treatment of consumption, especially sanatoria, had become general, and it was seen that many forms of tuberculous laryngitis, especially those which do not affect the upper aperture of the larynx, can exist without causing grave discomfort, and may well be left alone *in cases of advanced consumption.* I venture to think, however, that the reaction against active treatment went too far, and I hope to be able to show that a great deal of good can be done by active measures in really suitable cases.

### Cases Suitable for Active Treatment.

Tuberculous laryngitis is found more often than is generally realised at quite an early stage of consumption, and it attacks a very considerable number of the subjects of chronic phthisis who, but for this complication, have the prospect of many years of life and work. In such cases the laryngeal affection can, not infrequently, be healed by active treatment, often it can be improved and its progress stayed; and it must be remembered that death from pulmonary tuberculosis is far preferable to the very painful ending of the victim of advanced tuberculous laryngitis.

General treatment must in no wise be neglected; the sanatorium method, carefully carried out, is of the greatest value. It is in no way contra-indicated by laryngeal tuberculosis, but only by acute catarrhal laryngitis. It is important, however, in selecting a sanatorium to be convinced that the throat will be carefully observed and, if necessary, treated; and it is often advisable to proceed with the treatment of the larynx before sending the patient away. The healing of a well-marked case of tuberculous laryngitis by general treatment alone is an extremely rare occurrence and, on the other hand, I have seen cases heal under local treatment in the unfavourable surroundings of a hospital out-patient; general and local treatment together form the best possible combination.

### Some Results of Active Treatment.

The results obtained at the Mount Vernon Hospital show a proportion of successes which, though not sensational, prove, I think, that active treatment is of great value, and that more cases of tuberculous laryngitis can be healed and markedly improved by this method than by sanatorium treatment alone. This institution is managed as a hospital, not as a sanatorium, and many cases are admitted in an advanced stage of phthisis. Of 45 consecutive cases of undoubted laryngeal tuberculosis admitted in 1906, 20 were considered unsuitable for any attempt at local cure by active treatment; in nine of these operations with punch-forceps were performed for dysphagia, which was improved in every case and in most greatly relieved; of the remaining 25 cases, in which active treatment was carried out, six were healed and seven much improved.

It is unwise to send laryngeal cases abroad, for they may rapidly become worse at any time, and there is a general consensus of opinion that they are unsuitable for treatment at the high-level resorts of Switzerland. Complete rest for the voice, involving absolute silence and the use of a slate and pencil, has in many cases a great effect, especially in superficial lesions of the cords or interarytenoid space, and I have seen patients improve remarkably under this *régime.* But it is difficult to carry out even in a sanatorium and often has a depressing effect on the patient, so that it may be reckoned among the severer measures, only indicated where there is a real chance of doing good. Excessive coughing throws a great strain on the throat; the patient should be told of this danger, for he can often do much to restrain it by an effort of will. In cases of severe dry cough heroin is very useful; the dose should not be too small, $\frac{1}{12}$ to $\frac{1}{6}$ grain, and may be given in a lozenge or a linctus of glycerine and water; linctus containing sugar should be avoided as detrimental to the appetite.

### Early and Favourable Cases.

It follows from what I have said above, that the state of the pulmonary disease and of the general health must be the chief guide in deciding whether to treat actively the laryngeal complication, and I am accustomed to divide my cases, to make this principle clear, into three groups. In the first come those in which the lung disease is in a quite early stage and is not rapidly progressing and where the general health is good; cases in which, but for the laryngeal complication, the physician would now give a good prognosis. Of these cases one may say that a cure is possible and that the less disease is apparent in the lungs the more energetic should our treatment be. As a rule in these patients the laryngeal lesions are not of a severe type, but consist rather of superficial infiltration and ulceration on the cords or interarytenoid space, and can be healed by the proper application of pigments; but if the ulcers are deeper or more refractory, or if massive infiltration is present, no time should be lost in resorting to punch-forceps or curette.

### More Advanced Cases.

In the second group, a large class, are the cases in which the disease in the lungs is so far advanced that a definite cure cannot be expected, but is not rapidly progressing, and has not greatly affected the general health and the body weight; and even rather more advanced cases if they are quiescent or improving. In many of these cases, also, the larynx can be definitely healed; in many others much improved with a fair prospect of life and work for a considerable time. But cases in this group must be carefully chosen; it should be remembered that the physical signs of disease in the lung are often masked by the laryngeal affection, and the patient should be watched for a week or two, and the temperature and body-weight noted and considered, before a series of operations with punch-forceps is undertaken in the hope of curing the disease. If treatment be decided on, it should be thorough. The extent and situation of the laryngeal disease must be considered in these cases. Thus, the typical tuberculous thickening in the interarytenoid space, if it does not interfere with the apposition of the cords, causes no symptoms, often remains unchanged over long periods, and may very well be left alone if it can be watched. On the other hand, definite ulceration in this region tends to spread quickly to the arytenoids and so cause dysphagia; it should therefore be treated with pigments and curette Again, an ulcer or infiltration on the vocal cord damages the voice, distresses the patient, and interferes with his work; it also should be submitted to active treatment if the patient's general condition allows.

Infiltration involving only the interior of the larynx, and neither the epiglottis nor arytenoids, is generally more or less superficial and can often be healed by active treatment. I will describe two typical cases, in both of which healing was obtained in the out-patient department without the advantages of sanatorium treatment.

*Case I.*—Agnes W., aged 31, came to the out-patient department in November, 1903, suffering from hoarseness and almost constant aphonia of three years' duration; there was a diffuse red infiltration of the left cord with an ulcer at the centre and numerous interarytenoid granulations, and there were signs of phthisis at the apex of the left lung. The general health was fair, but she was nervous and intolerant. Pigments of lactic acid and formalin were applied, and, in June, 1904, the ulcer had healed, but the infiltration and the interarytenoid granulations remained and she was still aphonic. Lake's pigment was now used once a week, and in August the voice returned and became steadily stronger. By October only a slight swelling remained on the front of the left cord and the interarytenoid granulations were reduced to a slight uniform thickening; local treatment was discontinued, and, when seen in November, 1906, the larynx appeared normal and no thickening of the cord could be detected.

*Case II.*—Louisa H., aged 17, was seen in the out-patient department on August 2, 1906. She had had cough and profuse expectoration for 12 months, and had been hoarse for 10 weeks. There was somewhat extensive phthisis of both upper lobes and a cavity on the left side. The larynx showed typical granular swelling of both cords. The general health was moderate, but was decidedly improving, and for this reason I decided on treatment, and applied the pigment once a week. By August 30 the voice was almost normal, the cords were smooth, red, and thickened, and the granules had gone except for a small patch near the front of the right cord. A month later the cords, though thickened, were quite smooth and firm. She was now admitted to the hospital, and the local treatment was continued for a fortnight and then omitted. When she left the cords were normal but for a little cicatricial thickening, and when last seen, on January 31, 1907, the condition remained unchanged.

Considerable infiltration of the arytenoids or even epiglottis is in itself no bar to active treatment, as the following cases show; but very extensive disease, especially deep ulceration, rarely occurs unless the patient's resistance is completely broken down, and is not suitable for local curative treatment.

*Case III.*—Alice G., aged 21, was admitted in July 1905. She had chronic phthisis of both lungs, had been ill for six years, and had been hoarse for five weeks. Dysphagia had come on three weeks before, and was so severe that she was unable to swallow solid food; until this the general health had been fairly good. The epiglottis was greatly infiltrated and was ulcerated on the laryngeal surface; the rest of the larynx was normal—a very unusual combination. I removed the epiglottis in two pieces at different sittings, and applied Lake's pigment daily. The dysphagia was completely relieved from the date of the first operation, and a fortnight after the second the wound was healed. When seen in February 1906 the larynx remained soundly healed. She died from a profuse hæmoptysis in December 1906, but her mother informed me that up to the time of her death there were no throat symptoms and the voice was good.

*Case IV.*—Elizabeth P., aged 30, was admitted on May 23, 1905, with early phthisis, a history of hæmoptysis, and weakness of the voice of fifteen months' duration. The arytenoids, especially the right, were slightly swollen, the right cord red, and the interarytenoid space thickened. Lake's pigment was used daily, and on May 30 part of the right arytenoid was clipped away. The wound healed rapidly, and on July 5 no infiltration was visible in the larynx, a slight puckering marking the site of the operation, and when seen in February, 1907, the larynx remained sound.

Occasionally, much more extensive disease does well under active treatment:—

*Case V.*—Alfred P., aged 21, was admitted on August 15, 1905, with phthisis of both lungs, a cavity on the right side, hoarseness and aphonia of nine months' duration, and slight dysphagia. The vocal cords were much infiltrated, both arytenoids were considerably swollen, and the general health was poor. Under these circumstances active treatment was at first not considered advisable, but the dysphagia becoming worse, a piece was removed from the left arytenoid a week later. This was followed by a marked improvement, and on September 5 I noted "No dysphagia, the left arytenoid quite shrunken, so that the corniculum laryngis is visible." On September 26 a piece was removed from the right arytenoid, which had become more swollen. Lake's pigment was used in half-strength, and when the patient was discharged in November the arytenoids were not swollen, the left ary-epiglottic fold was slightly enlarged, there was some smooth interarytenoid thickening, the ventricular bands were slightly swollen, but the cords were normal. There was no dysphagia, and the voice was good. I heard five months later that the throat was giving no trouble. This is not a case of complete and definite cure, but at least there was marked improvement.

### Palliative Treatment in Hopeless Cases.

In the third group are the hopeless cases, which have no chance of restoration to any degree of health; cases with decided cachexia, hectic fever, rapid loss of weight, or acute or miliary tuberculosis. In all such cases the treatment must be purely symptomatic; when there are no severe symptoms it is cruel to trouble the patient with useless local treatment. The sensation of dryness and discomfort may be to some extent relieved by inhalations or, better, by a solution of menthol in liquid paraffin, 15 grs. to the ounce, in a good atomizer. Intratracheal injections of menthol, 1 per cent. in paraffin, are sometimes comforting to the patient.

If dysphagia supervene, much can be done by careful attention to the diet, which must be soft, unirritating, and not too hot. Semi-solids are usually tolerated better than solids or liquids, and for some reason milk is often an especial difficulty; junkets, meat-jelly, pounded raw beef-steak, raw eggs, oysters, and such like, may all be suggested as easy to swallow. Morphia is sometimes used as an insufflation in doses of $\frac{1}{6}$ gr. diluted with starch, or cocaine as a spray in 5-per-cent. solution; but these drugs should never be employed except in the last stages, for they quickly lose their effect and have a most disastrous result on the appetite and general condition of the patient. Orthoform is the best analgesic we possess for this purpose; it is used as an insufflation in doses of 3 to 5 grs. either alone or mixed with an antiseptic powder such as amyloform; its action usually takes about half an hour to develop fully, and it lasts for a long time; it is safe and may be entrusted to the patient, who can be taught to suck it into the larynx through a glass tube by the method of "auto-insufflation." A similar drug, anæsthesin, is also in use, but appears to me to be less effective.

In severe cases of dysphagia, in tuberculous laryngitis, however, all this treatment is but too often ineffectual, and in such patients removal of the infiltrated epiglottis with cutting-forceps or punching out even a small part of the swollen arytenoid may be relied on to relieve or even to abolish this most distressing symptom. The operation takes but a few seconds, it is almost painless after careful cocainisation, and causes so little disturbance that it may be performed on patients in the most advanced stage of phthisis. This method of treating the dysphagia has not received the attention it deserves, for the good effect is most remarkable. Again, if the arytenoid swelling be so great as to produce dyspnœa, removal of a small part will cause the whole mass to shrink and will obviate the necessity for tracheotomy. These wounds tend to heal in the most extraordinary manner, and hardly ever become inflamed, even in the most advanced cases; I have never seen the local condition made worse by such treatment.

*Case VI.*—T. K., a man of 31, with extensive phthisis of the left lung, came under my care in June 1904. He had been hoarse for four years, and had suffered from dysphagia for a month; this had been so severe for the last nine days that he could "only force down fluid food," and he had lost 6 lb. in weight in a fortnight. There was decided obstructive dyspnœa with inspiratory stridor. The arytenoids were enormously swollen, almost blocking the laryngeal aperture. I removed a piece from each with punch-forceps, and he returned next week very pleased with the result, for he had been able to eat meat immediately after, and had gained a pound. In a month he was at work and had no dysphagia. Three weeks later he had given up work and was short of breath; there was no laryngeal dyspnœa or dysphagia. From this time he quickly sank, and died three months after I first saw him. His father told me that his throat gave him but little trouble to the end, and there was no obstruction to breathing.

*Case VII.*—B. R., a white-haired man of 60, came to me on November 10, 1904, with hoarseness, moderate but increasing dysphagia for four months, and phthisis of both lungs with cavitation of the right side. As he was very feeble I tried to avoid surgical treatment, and gave insufflations of orthoform; but the dysphagia became severe, and I admitted him and removed the infiltrated epiglottis on December 20 with great and immediate relief. On February 16 there was only slight discomfort, and he was "going to resume his work"; however, he became weaker and died in March, but had no severe dysphagia to the end of his life.

Such results are by no means exceptional, but are indeed the rule where severe dysphagia is due to tuberculous infiltration of the upper aperture of the larynx.

### Technique of Active Treatment.

Finally, I will shortly describe the technique of the methods of active treatment which I am in the habit of employing. And from this point of view the cases of tuberculous laryngitis may be divided into two main classes, those involving the upper aperture of the larynx and those which affect only the interior of the organ. The former are of by far the most serious import, for it is only here that tuberculous infiltration will cause severe dysphagia, and here—the submucous tissue being plentiful and loose—that "massive" infiltration occurs beneath unbroken epithelium.

For superficial lesions, such as occur commonly on the cords and interarytenoid region, I employ a pigment introduced by Lake, containing lactic acid 50 per cent., formalin 7 per cent., and carbolic acid 10 per cent.; the latter has a distinctly analgesic action, and pain rarely lasts for more than a few minutes after the application. This solution is applied on a strong cotton-wool mop, and must be well and firmly rubbed in; a light application is useless. It may cause healing when used only once a week (see Cases I. and II.), but as a rule I get a better effect by daily applications. If the resulting reaction is too great, the solution may be diluted or applied less frequently, and it is always better to do this before leaving off the treatment altogether after healing has occurred. In the treatment of ulcers which do not improve, or of sprouting granulations in the interarytenoid region, I find Heryng's curettes of value; the object is to remove the part cleanly in one piece, and some degree of force is required. But these methods are quite ineffectual for the treatment of massive infiltration lying beneath unbroken epithelium, such as occurs on the arytenoids and epiglottis. For this purpose punch-forceps are necessary, and I prefer Lake's pattern for the arytenoids as being very powerful and effective. To remove the free part of the epiglottis I use a large punch-forceps of my own pattern; it will readily excise the organ at one bite, a very considerable advantage over other and smaller patterns, with which two or three separate punchings are necessary. There need be no hesitation about using punch-forceps for fear of converting a closed infiltration into an ulcer, for these wounds never seem to become inflamed. In very advanced cases, when the operation is done to relieve dysphagia, the wounds do not always completely heal, though they usually do so even then in the arytenoid region; but this is of little importance since the dysphagia is relieved. In all other cases the wound heals with great rapidity, even when the infiltration is not completely removed. The swollen arytenoids will generally be found to shrink rapidly after the first operation, and less will have to be done than appeared probable at first; but all the swollen parts should be removed as thoroughly as possible, and one should do as much as one can at a sitting. The larynx must be made thoroughly insensitive by 10 per cent. cocaine or alypin, applied with a drop-syringe; in this way the larynx is not irritated. The cocaine is applied only where it is wanted, and there is little risk of toxic symptoms.

Though, as tuberculous laryngitis is but a complication of pulmonary phthisis, its treatment will never, on the whole, be brilliantly successful; yet I hope to have shown that active measures used with discretion are of great value, both in relieving the terrible dysphagia of advanced cases and in procuring arrest and healing in the earlier stages. The moral of this is to see that cases are treated in the early stage; too often do cases present themselves for the first time when the disease in both larynx and lungs is hopelessly advanced. If it be remembered that this complication occurs quite often in an early stage of phthisis, and that it may cause no symptoms whatever, then it will be clearly seen that the only way to improve the results of treatment is to examine the larynges of all cases of consumption at regular intervals as a matter of routine. At the Mount Vernon Consumption Hospital the throat of every in-patient is examined on admission, and out-patients are sent to the throat department if a history of the slightest throat trouble can be elicited. In this way I am often able to cure in quite an early stage patients who would probably have allowed the disease to become extensive before any local symptoms attracted their attention.

